# Trauma Exposure Checklist: Preliminary Results Show Promising Psychometric Properties to Assess Subjective Perceptions of Exposure to Potentially Traumatic Events

**DOI:** 10.3390/bs14100892

**Published:** 2024-10-02

**Authors:** Alice Murteira Morgado

**Affiliations:** Centre for Research in Neuropsychology and Cognitive Behavioral Intervention, 3000-115 Coimbra, Portugal; alicemmorgado@gmail.com

**Keywords:** trauma exposure, lifespan development, social support, loss, checklist

## Abstract

Most individuals in the general population will be exposed to potentially traumatic situations at some point in their lifespan. There is a strong body of research focusing on the clinical implications of exposure to potentially traumatic situations, but less attention has been given to psychological adjustment following these events. Very few available instruments assess trauma exposure while considering subjective perceptions of both positive and negative features. In this study, 304 adults from the general population, mostly women of middle and high socioeconomic status (mean age = 43), filled in a questionnaire with sociodemographic questions and an adapted version of the Trauma Exposure Checklist, which was originally developed in the context of the COVID-19 pandemic. Results indicate that most participants have experienced a potentially traumatic event, most prevalently related to violence and/or criminality and to the severe illnesses and deaths of close relatives. Principal components and reliability analyses lent support to a three-factor structure that considers perceptions of internal and external losses and a positive factor that addresses resources and social support. This study is a first step towards advancing our knowledge on the impact of developmental and sociodemographic factors on subjective experiences of psychological crises and, consequently, psychological adjustment throughout the lifespan.

## 1. Introduction

Potentially traumatic events may be defined as crisis-triggering events that can be perceived as uncontrollable and involve significant human suffering, affecting one’s ability to function normally for some time [1,2]. During their lifespan, most of the population will be exposed to potentially traumatic situations [3,4,5,6]. These situations may include crisis-triggering events, such as war and forced relocation [7], criminality [8], natural and human-made disasters [9], or other extreme events, but they may also include relatively common events that involve social and/or psychological hardship, such as grieving the death of family member [10], family changes [11], and poverty [12], among others. In fact, many human experiences, besides those that are included within clinical definitions of trauma (e.g., the DSM-5 definition of trauma), have been found to produce potentially traumatic responses [13,14,15]. The type, duration, location, severity, cultural context, and political background of these experiences may influence the impact on individuals of different developmental stages, sex, ethnicity, socioeconomic status, and sociocultural conditions [7,16].

There is a strong body of research focusing on the clinical implications of exposure to potentially traumatic situations [17,18] and especially on psychopathological outcomes and symptom relief [19]. As crucial as this is to tackling psychopathology in a high-risk context, those who do not develop psychopathological symptoms should not be overlooked following potentially traumatic situations as their psychological adjustment may still be compromised [18,20,21], and these experiences may affect most individuals at some point in their development. This study addresses this gap by looking at how potentially traumatic situations may involve both gains and losses in different developmental stages and how they may apply to the general population rather than just those who develop psychopathological symptoms. This involves considering both individual psychological factors and community-level resources [7,20] and looking at diverse groups to check for potential developmental, gender, and cultural differences in disaster exposure experiences [2].

More than their objective characteristics, these experiences entail significant challenges to the psychological adjustment of individuals in all life stages and in all their life contexts due to its subjective impact [5,7,20,22]. Individuals may respond differently to potentially traumatic events [5] and go on to follow different pathways, with some being affected by psychopathological symptoms and others going on to follow resilience and recovery trajectories [23]. Some research has focused on positive responses to adversity, including resilience and growth following adversity [15,24]. Research on post-traumatic growth highlights how extremely adverse experiences may result, for some individuals, in more adaptive functioning levels and how this is a common experience that should not be overshadowed by psychopathological symptoms but should instead be considered in relation to them [20,22,24,25,26,27]. Indeed, these contributions support the relevance of looking at both negative and positive consequences in order to understand trauma and adversity throughout development in the most comprehensive way possible.

Overall, this suggests that research should be looking at a wider scope of consequences rather than limiting the focus on the presence or absence of psychopathological symptoms. However, within the considerable number of measures to assess trauma exposure [18], most of the available instruments focus on psychopathology (outcomes and prevention). This is another piece of evidence concerning how available measures have been overlooking the fact that exposure to traumatic events does not always result in psychopathology [23,24,25,26,27]. Indeed, very few available measures focus on both the positive and negative consequences of exposure to a traumatic event, and even fewer have considered individual meaning for one’s life [18]. Despite its recognized role in psychological adjustment following exposure to traumatic events [15], social support has also been overlooked in research on trauma exposure [16].

A substantial body of literature has suggested that more than the event itself, it is important to look for the individual meaning and consequences (on physical, individual, and social levels) it entails for each individual [2,5,13,14,20,28], depending also on their developmental stage [15,20,29] and ecosystems, including the communities that they are a part of [16,22,30]. In particular, the role of social support has been highlighted as one of the most important protective factors for psychological adjustment after exposure to traumatic events [7,16].

The COVID-19 pandemic, with its unique features and implications, provided a recent and unprecedented opportunity to study the impact of a crisis-triggering event in the general population. The fact that it was experienced worldwide and involved severe unforeseeable consequences at many levels elicited strong research efforts across the globe to study its psychological and social impact [31,32] from many different perspectives (occupational, clinical, educational, social, etc.). In this scope, a research project involving the Portuguese Population [1] developed a psychometric tool with which to assess the degree of exposure to the COVID-19 pandemic, conceptualized as a potentially traumatic event: the Trauma Exposure Checklist (TEC). This checklist was based on the conceptual model of Morgado (2020) [20] and considers individual and context-related stressors together with protective factors in crisis situations to assess how individuals perceive two major dimensions: exposure to trauma and support from the community.

The TEC validation study [1] supported the robustness of this instrument in assessing COVID-19-related protective and risk factors within individual and community levels, not focusing exclusively on individual psychopathological symptomatology [33,34] but, instead, on individual meaning concerning negative and positive outcomes. The original version of the TEC includes two self-report scales to assess self-perception of exposure to trauma and perceived community support during crisis, showing good psychometric properties within the context of COVID-19, and it was used to study the impact of the initial stages of confinement in Portugal [35]. The results suggested that those living alone, young adults, and women were relatively more vulnerable to the impact of COVID-19 confinement experiences as they showed higher levels of exposure to trauma and lower quality of life. For example, younger adults (18–24 years old) showed poorer psychological quality of life scores and perceptions of support compared to older age groups. There were also differences between young adults and middle-aged adults in terms of coping strategies and quality of life [36].

These results support the relevance of a lifespan developmental perspective for the study of potentially traumatic events [15] as they suggest that adults have different perceptions of exposure to these events and attribute different meanings to such experiences, based, among other factors, on their life stage and the respective gains and losses involved [15,37], as well as their orientation towards growth vs. maintenance [38]. For example, although young adults were at lower risk for COVID-19 symptoms, they may have been more psychologically affected during confinement due to the loss of social relations and greater isolation, which disrupted their routines, developmental expectations, and way of life to a greater extent when compared to middle-aged and older adults [35].

Having overcome the COVID-19 pandemic, the presented research consists of an adaptation of the TEC outside this context and within a broader scope of experiences. This study aims to respond to the identified gaps in the literature by testing the psychometric properties of a new version of the TEC. The aim is to provide a robust measure of exposure to multiple potentially traumatic events that considers subjective perceptions of losses and support in individuals not affected by psychopathology and in different developmental stages.

The following research questions were considered:

1. What types of experiences do people perceive as potentially traumatic events?
2. Can the TEC be adapted to encompass a wide range of potentially traumatic events?

This study reports reliability and validity assessments of this psychometric tool and describes the types of events classified by participants as potentially traumatic. In addition, it reports the main scores of the sample (means, std deviations) based on gender, socioeconomic status, age of exposure to event, and current developmental stage.

## 2. Materials and Methods

The sample was formed out of voluntary participation, resulting ina convenience sample of adults (N = 305) from 18 to 92 years old (mean age = 43.15) from the general population of individuals living in Portugal. The survey was advertised online via social media and through flyers that were distributed in community centres, universities, and social/commercial locations. Individuals had the option of filling in an online questionnaire or a paper questionnaire. The latter were provided in community centres, mostly for elderly participants who were not comfortable with online surveys. These individuals had the opportunity to clarify any doubts with a research assistant that was pre-sent for some periods in these community centres, making sure that interested participants had the sufficient conditions to respond individually and anonymously to the questionnaire. No adverse reactions to the questionnaire were reported.

Most participants were females (81%) of medium (53%) and high (39%) socioeconomic status. Participants were included in one of three study groups—“young adulthood” (25%), “mid-adulthood” (63%), and “older adulthood” (13%)—based on a combination of several developmental indicators. In addition to chronological age, participants’ developmental stage was based on a combination of several sociodemographic indicators, namely, occupational status, marital status, and caring responsibilities for others. Developmental stage was coded as [Young adult = 1; Middle-aged adult = 2; Older adult = 3]. For each participant, this was determined via their average score on the following indicators as coded: chronological age [18–30 = 1; 30–64 = 2; 65+ = 3]; occupational status [student = 1; active/working/unemployed = 2; retired = 3]; marital status [single = 1; married/civil partnership/divorced/separated = 2; widowed = 3]; and caring responsibilities [no caring responsibilities = 1; siblings or children = 2; grand-children/parents/spouse = 3].

Indeed, as much as chronological age may be a common and important indicator, it is widely accepted that developmental stage is not limited to the number of years one has already lived. As Infurna [15] suggests, “development is a complex and heterogenous process that cannot be explained solely by chronological age” (p. 808). It is important, therefore, to consider gains, losses, responsibilities, and, in particular, resources that are developmentally specific [38] when defining the different stages of adulthood [39].

The sample distribution is summarized in Table 1.

### Data Was Collected through Two Self-Report Questionnaires

The sociodemographic questionnaire included questions on age, gender, socioeconomic background, family structure, family responsibilities, and occupational status.

The Trauma Exposure Checklist was adapted from the original version developed in the COVID-19 pandemic context, as mentioned above. Originally, the TEC [1] included 24 items, divided into 2 parts: individual perceptions (losses [α = 0.833] and resources [α = 0.898]) and support measures (global measures [α = 0.674] and local measures [α = 0.939]). Items were answered on a 6-point Likert scale.

For this study, a definition of potentially traumatic events was provided (“critical events outside one’s control that involve significant suffering and affect one’s ability to function normally on everyday life for some time”), and participants were asked to identify personal past experiences that fit within this definition (including the types and timing of these experiences) and to classify the consequences and impact into two dimensions based on the initial version of the first part of the TEC: losses and resources.

The original items were used and adapted to address a broader scope (i.e., more than the challenges that were specific of the pandemic context), resulting in a structure of 24 items, including all those from the original version (13 items about losses following exposure to potentially traumatic experiences and 11 items about resources available in the same context). A 5-point Likert scale was implemented to allow for a neutral response option.

The proposed adaptations were then evaluated by a group of experts composed of psychologists and academics within the fields of developmental and clinical psychology, who assessed the adequacy and relevance of each item and provided additional suggestions. As a result, 5 items were rephrased, 1 item was removed, and 1 new item was included. This adapted structure was then tested by a group of volunteers composed of 34 BsC psychology students, confirming promising preliminary internal consistency [losses (13 items): α = 0.721; resources (11 items): α = 0.873].

Before data collection, the study was evaluated and approved by the ethics committee of the researcher’s academic institution.

Participant recruitment was undertaken online and through institutional invitations/announcements, as stated above. Prior to accessing the protocol, participants were provided with an informed consent form, where they were debriefed about the aims of the study and the exclusion criteria. The only preconditions for participation were being an adult (aged 18+) and not having a diagnosis of psychopathology at the time of the participation.

Participants were provided with a full description of the objectives, institutional framework, length, and confidentiality issues. It was stated that participation was voluntary, with no incentives for participation being offered. No information that would allow us to identify each participant individually was requested, thus granting the anonymity of all data. Participants were assured of the confidentiality of the study and informed that their participation was voluntary. Items completion took around 10 min.

After consenting and confirming that no exclusion criteria was applicable, individuals were either forwarded to an online form where they registered their answers anonymously or invited to fill in a paper form, also anonymously.

Data were inserted and analyzed on IBM SPSS statistics v.27. Descriptive statistics were performed to explore the distribution of responses relative to exposure to potentially traumatic events.

To confirm the psychometric robustness of the adaptations made to the TEC, items on each scale were analyzed by examining the distribution of different responses, inter-item correlations, and the correlations of each item with its corresponding subscale. A principal component analysis with varimax rotation was performed to check the adequacy of the items for each factor. Mean scores were also calculated considering several sociodemographic, developmental, and exposure factors.

## 3. Results

### 3.1. Descriptive Statistics—Exposure to Potentially Traumatic Events

Exploratory questions included in the TEC allowed us to collect some relevant information about the types of events participants consider to be potentially traumatic. In fact, given the definition, i.e., “critical events outside one’s control that involve significant suffering and affect one’s ability to function normally on everyday life for some time”, 198 participants (65%) identified experiences that affected them directly or indirectly and fit within this definition. Of those affected, 122 (62%) reported having been exposed to two or more events over their lifespan. In these cases, the questionnaire instructed participants to refer to the most significant event they had experienced while answering the remaining questions of the TEC.

The types of events, age at time of initial exposure, and timing of exposure are presented in Table 2.

As shown, the most reported type of event was violence or criminality, including experiences of domestic violence, sexual abuse, and bullying. Severe illness was also a frequently reported type of event, including physical and mental illnesses of participants or of their close relatives. The third most reported type of event involved the death of a close relative. Among other types of events, participants reported war and drug addiction on the part of those close to them, for example. Regarding timing of exposure to the event, most participants referred to childhood and adolescent experiences (N = 73) that occurred more than 10 years ago (N = 91).

### 3.2. Principal Components Analysis

Of the 198 participants that reported exposure to a PTE, 4 did not complete the 24 questions of the TEC. All 24 items of the Trauma Exposure Checklist (adapted version) were subject to principal component analyses with varimax rotation, considering all complete responses to the instrument (N = 194).

Four basic criteria were followed to retain and interpret the factors: (a) changes in eigenvalues represented in the scree plot; (b) component loadings equal to or greater than 0.40; (c) the presence of the underlying theoretical referent for each component; and (d) an internal consistency reliability of 0.70 or greater [40]. The suitability of the intercorrelation matrix for factor analysis was demonstrated by low-to-moderately high inter-item correlations (0.01–0.67), with higher inter-items correlations found for items in the same scale, a strong KMO (0.79), and a significant Bartlett’s test of sphericity (Χ2 [276] = 1973.40, *p* < 0.001) [41].

Principal components analysis with varimax rotation and auto values greater than 1 revealed three contributing factors to explain the 45.27% variance of the data. Six items loaded saliently on the first factor, namely, those related to individual psychological losses associated with exposure to a potentially traumatic event (Losses—Internal), for example, threats to one’s life, changes in personal routines, etc. Six items loaded saliently on the second subscale, all corresponding to losses of others or material losses (Losses—External), for example, witnessing threats to other’s well-being, losing objects or property of material or affective value. The third subscale had 11 items loading saliently. These items referred to sources of social and emotional support and resources that emerge when facing a crisis (“Support”). One item (“grief or deep sadness for a loss”) was excluded due to loadings bellow 0.40 in all three factors.

### 3.3. Reliability Analysis

The results of the Trauma Exposure Checklist analysis of internal consistency for this sample were 0.719 for the component “Losses—Internal” (6 items); 0.744 for the component “Losses—External” (6 items); and 0.882 for the component of “Support” (11 items). These results confirm good internal consistency for the three subscales. In both cases, item total statistics indicated that excluding any of the items would have not improved internal consistency. All subscales meet the threshold of acceptability of 0.65 proposed by DeVellis [41]. This means that the items of the three subscales represent a good measure of the construct of exposure to potentially traumatic situations in this sample.

The structure matrix of the 2024 version of the TEC, with its corresponding alpha coefficients, is presented in Table 3.

Table 4 presents the mean scores in all three TEC factors considering developmental stage, gender, socioeconomic status, type, and timing of event. These are based only on descriptive analyses of means and standard deviations as the number of participants and size differences between groups did not allow for inferential analyses.

Descriptive statistics show that females presented lower scores in terms of internal losses and support, but higher scores for external losses compared to males.

Individuals with medium SES showed lower internal losses, while those with low SES showed higher perceptions of support.

Younger adults perceived higher internal losses, while middle-aged adults perceived higher external losses and older adults perceived higher support following exposure to PTE.

The type of event associated with higher perceptions of internal losses was natural disaster, whereas external losses were perceived as higher for those who have experienced violence/criminality. Support was higher for those who experienced accidents.

As for the age and timing of exposure, internal losses were higher for those who experienced a potentially traumatic event at 19–35 years old and within the last 2 to 5 years. External losses were higher for those exposed at older ages (66+) and experiencing continued trauma. Perception of support was higher for those exposed between 36–65 years old and having experienced trauma within the last year.

## 4. Discussion

The present study aimed to answer two research questions:

1. What types of experiences do people perceive as potentially traumatic events?
2. Can the TEC be adapted to encompass a wide range of potentially traumatic events?

Regarding the first question, it was clear that many different experiences were considered potentially traumatic events by this sample, which, as previously mentioned, consists mostly of women. This supports previous research that has challenged clinical definitions of trauma-triggering events [13,14,15]. For example, divorce and family challenges (conflicts, complications with pregnancy and birth) were mentioned by several participants as potentially traumatic events. Although these results were somehow expected, they further support the literature that highlight the importance of looking beyond clinical definitions of trauma [13,14,16], providing more evidence that common events—which are less severe and pathological than clinical definitions of trauma—may as well be considered by individuals as potentially traumatic and should not be overlooked by relevant health and social support services. Also noteworthy was the fact that most reported experiences occurred during childhood and/or adolescence and happened more than 10 years ago. Still, they are viewed as potentially traumatic for this sample later in life, even if they have not resulted in psychopathology. This finding supports the research and intervention endeavors to address these experiences and promote psychological adjustment throughout the lifespan [18,20,21].

Considering the second question, it was possible to adapt the TEC in order to assess individual reports of different potentially traumatic experiences consistently in a way that is reliable and relevant to screen for risk and protective factors. A three-factor structure with good reliability scores was obtained. It includes two dimensions of loss (internal and external) and one dimension of support (social support and resources and positive connections). Indeed, these results appear promising for the general population; however, they should be interpreted with caution when applied the male population, as most of this sample was composed of women (81%).

Although in the original version of the TEC (which applied specifically to the COVID-19 pandemic as a potentially traumatic event), the factor “Losses” was a single factor, in this adaptation, the items concerning losses loaded saliently in two different factors. Upon analysis of which factors loaded more saliently in each group, it was evident that one referred to personal, individual, “Internal Losses”, including threats to the self, and changes to one’s life, while the other referred to “External Losses”; that is, losses that were less directed towards the self and more related to external elements (either other people or objects/possessions). This is an interesting finding which may show how internal and external losses may have different meanings for individuals experiencing potentially traumatic events. However, it could also be partially explained by specific perceptions and experiences based on cultural aspects attached to female gender roles, given the representativity of women in the sample, which is why further validation of the TEC needs to be conducted with a more robust sample that includes a balanced number of males and females.

The factor “Support” includes social support, a dimension that has been somehow ignored in research on trauma exposure [16]. This factor highlights the importance of individuals’ perceived support and emotional connections to significant others and to valued resources of the community, such as work, school, organizations, neighbors, etc., in the context of crisis and adversity [20].

In sum, the TEC includes what may be more and less significant to individuals following a potentially traumatic experience, allowing for the identification of the consequences that are more valued by individuals. This includes negative impact (for the self or for valued external elements), which may place individuals at higher risk for poorer psychological adjustment trajectories, and positive impact in terms of social and emotional support, which may result in positive psychological adjustment throughout the lifespan. This is a first indication that the TEC may respond to a gap in available measures as it focuses on both the positive and negative consequences of exposure to a traumatic event and assesses subjective impact for one’s adjustment [18].

This study presents a preliminary assessment of an adaptation of the TEC to encompass a broader scope of experiences beyond the COVID-19 pandemic, focusing on subjective perceptions of exposure to potentially traumatic events. As this is an initial adaptation of the measure, some important limitations should be acknowledged and overcome in a future validation study. First, as previously mentioned, this study was mostly conducted with a convenience sample, mainly comprising women of middle and high socioeconomic status (a profile that is typically more available to participate in this type of study). Although women are more vulnerable to PTSD symptoms following exposure to trauma [6,42], caution is required concerning generalization to the overall population since sociodemographic characteristics, including gender and socioeconomic status (among others), have been found to predict the likelihood of being exposed to different types of PTE [6,42,43]. Likewise, although 304 responses have been registered, only 194 TEC questionnaires were completed, which limited the ability to perform other types of psychometric and inferential analyses.

Still, this research offers an important contribution to the study of trauma and loss as this adapted version paves the way for further research on gender-specific experiences and different developmental stages and on the impact of different relevant developmental and sociodemographic factors on subjective experiences of psychological crises. As such, future studies need to be conducted to support the robustness of the psychometric characteristics of this measure with a representative sample of the population. It is also important to carry on studies that overcome current limitations and include a more balanced number of participants considering important sociodemographic variables such as gender, socioeconomic status, and cultural background. This will not only allow for a stronger generalization of results; it will also allow for inferential analyses to be performed in order to compare scores based on sociodemographic variables. It will be important to evaluate how the TEC results can be impacted by several developmental factors and how they correlate and/or predict other relevant constructs, including quality of life, post-traumatic growth, and meaning in life.

## Figures and Tables

**Table 1:** Sample distribution.

		Frequency
Gender	Male	55
	Female	248
	Non-binary	1
Socioeconomic Status	Low	23
	Medium	160
	High	118
	Missing information/Not determined	4
Developmental Stage	Young adulthood	75
	Middle adulthood	190
	Older adulthood	39
	Missing information/Not determined	1

**Table 2:** Characteristics of events classified as potentially traumatic.

		Frequency
Type of event	Natural disaster	4
	Accident	24
	Violence or criminality	53
	Severe illness	46
	Others	66
	Death of close relative	28
	Family conflict/divorce/separation	8
	Pregnancy loss/interruption; birth complications	6
	Others/Not determined	24
Age at time of exposure	0–11 years old	39
	12–18 years old	34
	19–35 years old	61
	36–65 years old	55
	66 or more years old	4
Timing of exposure	Last year	13
	2 to 5 years ago	35
	5 to 10 years ago	26
	More than 10 years ago	91
	Continued trauma	18

**Table 3:** Trauma Exposure Checklist (24): 23-item structure matrix.

Items	Factor			Alpha Coefficient
	Losses—Internal	Losses—External	Support	
Threats to my life or physical integrity	0.783			0.719
Disease/Physical injuries to myself	0.550			
Psychopathology/Psychological injuries to myself	0.461			
Threats to my physical well-being	0.823			
Threats to my psychological well-being	0.684			
Sudden and negative changes in my routines	0.429			
Witnessing other people’s death		0.604		0.744
Witnessing threats to others’ life		0.703		
Witnessing others’ disease/injuries		0.781		
Witnessing threats to other’s psychological well-being		0.731		
Loss of goods or property with significant material value		0.486		
Loss of goods or property with significant affective value		0.501		
Strong and positive connections to my family			0.737	0.882
Strong and positive connections to my friends			0.757	
Strong and positive connections to my neighbours and/or community			0.756	
Strong and positive connections to my school and/or university and/or workplace			0.728	
Support from my family and/or close friends			0.655	
Perception of myself as someone who can handle crises well			0.492	
Support from my neighbours and/or community			0.767	
Support from my school and/or university and/or workplace			0.758	
Support from organizations and/or local institutions			0.635	
Support from emergency services			0.438	
Support from public services (Health, Justice, Social)			0.514	

**Table 4:** Mean scores in TEC.

		Losses—Internal		Losses—External		Support	
		Mean	Std. Dev	Mean	Std. Dev	Mean	Std. Dev
TOTAL		2.36	1.03	2.78	0.95	2.50	0.93
Gender	Male	2.44	1.13	2.69	1.13	2.70	0.92
	Female	2.33	1.02	2.80	0.92	2.46	0.93
Socioeconomic Status	Low	2.07	0.95	2.88	1.22	2.63	0.94
	Medium	2.47	1.07	2.88	0.98	2.55	0.94
	High	2.23	0.98	2.62	0.84	2.42	0.93
Developmental Stage	Young adulthood	2.45	1.07	2.74	0.98	2.35	0.75
	Middle adulthood	2.34	1.02	2.80	0.88	2.51	0.95
	Older adulthood	2.29	1.06	2.74	1.18	2.65	1.07
Type of event	Natural disaster	3.46	1.25	2.04	0.58	2.36	0.48
	Accident	2.26	0.97	2.72	0.81	2.92	1.05
	Violence/Criminality	2.27	1.12	3.26	0.93	2.21	0.90
	Severe illness	2.51	1.04	2.67	1.06	2.80	0.94
	Other	2.32	0.95	2.61	0.82	2.37	0.83
Age at time of exposure	0–11 years old	2.17	1.13	2.86	0.91	2.17	0.79
	12–18 years old	2.27	1.04	2.98	0.97	2.33	0.90
	19–35 years old	2.59	1.04	2.69	0.92	2.60	0.91
	36–65 years old	2.33	0.95	2.75	1.00	2.73	1.02
	66 or more years old	2.33	0.94	3.04	1.14	2.50	0.76
Timing of exposure	Last year	2.41	0.87	2.88	0.81	2.82	1.08
	2 to 5 years ago	2.55	1.07	2.89	0.91	2.27	0.71
	5 to 10 years ago	2.16	1.05	2.60	1.05	2.27	0.79
	+than 10 years ago	2.33	1.07	2.74	0.95	2.59	1.01
	Continued trauma	2.45	1.06	3.08	1.06	2.80	0.89

## Data Availability

The datasets generated during and/or analyzed during the current study are not publicly available due to further data analysis in the scope of the ongoing project. Datasets may be made available in the future from the corresponding author on reasonable request.
